# Ventriculoperitoneal Shunt Migration Into the Transverse Colon: A Case Report

**DOI:** 10.7759/cureus.52334

**Published:** 2024-01-15

**Authors:** Ahmad R Awwad, Manar S. H. Odeh, Diya Asad, Basel Y. M. H. Abu Rmeileh, Aya M. M. Dweik, Saja N. M. Baraka, Malek A. F. Karami, Iyas I. M. Awad, Afnan W. M. Jobran

**Affiliations:** 1 General Practice, Rafidia Governmental Hospital, Nablus, PSE; 2 Medicine, Faculty of Medicine, Al-Quds University, Jerusalem, PSE; 3 Medicine, Faculty of Medicine, Al Quds-University, Jerusalem, PSE

**Keywords:** neuro-surgery, pediatric, case report, transverse colon, vp shunt complication

## Abstract

A ventriculoperitoneal (VP) shunt is a connection between the cerebral ventricles and the peritoneal cavity. One of the rare complications of this procedure is shunt migration and perforation of the bowel. Our case report presents the case of a 19-month-old male patient who underwent VP shunt insertion due to hydrocephalus at the age of 8 months. He suffered from two episodes of bacterial meningitis at the ages of 11 and 15 months, requiring hospital admission. The patient's parents brought him to the emergency department after noticing a blood-stained diaper and seeing a part of the shunt extruding from the anal opening. Upon physical examination, the patient was active, neither in distress nor tachycardic. with unremarkable abdominal examination and negative peritoneal signs. A digital rectal examination showed normal anal tone, with normal-coloured stool with no blood at the tip of the finger, together with a compressible VP shunt.

Complications of this type of migration include faecal contamination and possible infections such as ascending meningitis. This case report highlights the extrusion of the shunt through the anal orifice in a 19-month-old male patient which serves as an example of the uncommon but serious consequence of VP shunt insertion in the pediatric population. While VP shunt insertion remains a widely used and effective treatment for hydrocephalus, healthcare providers need to recognize and address potential complications associated with this procedure. Additionally, this case emphasizes the importance of diligent monitoring and regular radiographic imaging to confirm the correct positioning of shunt components, particularly in the paediatric population.

## Introduction

Ventriculoperitoneal (VP) shunt insertion is the most widely performed modality in the treatment of hydrocephalus (HDC), which is a condition that occurs in the brain due to either decreased absorption or increased production of cerebrospinal fluid (CSF) that results in the dilatation of the ventricular system [1]. A VP shunt is a CSF diversion device, usually a tube, with a pressure-regulating valve that begins in the ventricular system [2]. Such devices aim to relieve excess intracranial pressure (ICP) by carrying CSF into an absorptive surface extracranially, such as the peritoneum [1]. Unfortunately, despite many advances in other fields of medicine, rates of shunt-related complications have remained the same at around 30%, with an overall long-term shunt failure rate reaching 46% [2]. The most common complications are shunt infection, obstruction, segmental disconnection, CSF pseudocyst formation, allergy, and shunt migration [3].

Perforation of the bowel caused by VP shunt is a rare complication with an estimated incidence rate of 0.1% to 1.0% among all cases of VP shunt displacement [4]. Most bowel perforations by the distal catheter occur in children, and protrusion of the tip of the catheter through the anus is the most common presentation. Among the children who have bowel perforation, <25% of patients have signs of peritonitis [5]. The exact risk factors and pathophysiology of such complications are still unknown. However, multiple hypotheses have been suggested. It could be attributed to the type of catheter used, such as silicon catheters [5]. A local inﬂammation and fibrosis at the contact site with silicon in the shunt tube or the CSF pulsations cause a continuous water hammering effect that can perforate the bowel wall, after which the shunt will continue through the lumen by the peristaltic waves [3].

In this case report, the authors present the case of a 19-month-old male patient in whom the distal end of the shunt had migrated and extruded through the anus.

## Case presentation

We present the case of a 19-month-old male patient who underwent a VP shunt placement due to congenital communicating hydrocephalus at the age of 8 months after being admitted due to a bulging anterior fontanelle and a low-grade fever. Since the VP shunt insertion, the patient has been admitted twice as a case of bacterial meningitis at the age of 11 and 15 months, for which he received intravenous antibiotics, as per CSF culture reports. During his second meningitis episode, the VP shunt was hardly compressible, thus, a brain CT scan was done which showed ventricular dilatation, suggesting VP shunt malfunction. As a result, the patient underwent VP shunt revision surgery and was discharged in good general condition.

The baby was brought to the emergency department by his parents after the visualisation of the VP shunt from the patient's anal opening and blood-stained diaper, and they took a photo of the situation. At the time of clinical evaluation at the hospital, the shunt was not visualised at the anal opening, however, there were dried spots of blood on his diaper.

Upon arrival, the patient was active, neither in distress nor tachycardic. His abdominal examination was unremarkable, with no peritoneal signs. A digital rectal examination showed normal anal tone, with normal-coloured stool with no blood at the tip of the finger. The VP shunt was compressible. Laboratory blood tests revealed a haemoglobin level of 8.6 g/dL, a mean corpuscular volume of 52.9 fL, and a hematocrit level of 28.7%. CSF analysis showed white blood cells of 300 (normal level 0-6 x 10ᶾ µL), neutrophils 60%, lymphocytes 40%, glucose 29 mg/dL, and protein 192 mg/dL. Abdominal ultrasound showed a gaseous abdomen with no evidence of peritoneal free fluids. 

**Figure 1:**
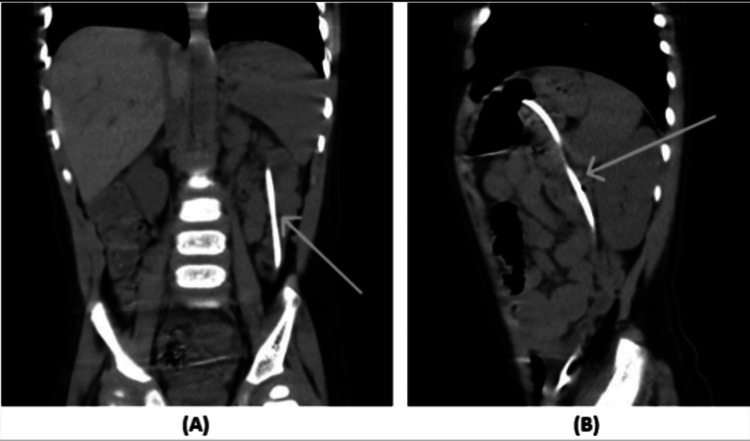
Plain abdominal CT scan (A) Coronal and (B) Sagittal sections showing the ventriculoperitoneal shunt invasion through the transverse colon.

A plain abdominal CT scan revealed intra-luminal migration of the VP shunt through the transverse colon, reaching the sigmoid colon, with no pneumoperitoneum or peritoneal free fluid (Figure 1A-1B).

The patient was prepared for surgery, and an exploratory laparotomy was done. Intra-operatively, exploration of the VP shunt at the temporal area of the skull was done. The old VP shunt was removed, and an extra-ventricular drain was placed.

**Figure 2:**
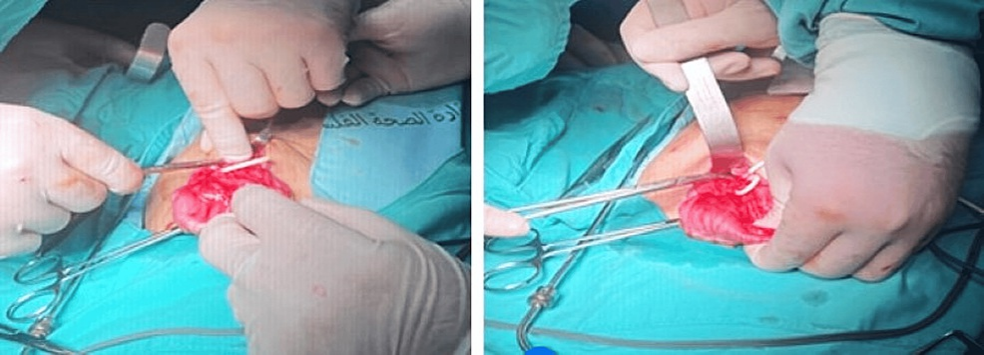
Intra-operative imaging showing the perforation of the transverse colon by the ventriculoperitoneal shunt

A right abdominal para-median infra-umbilical incision over the old VP shunt revision scar was done. We opened the abdominal wall without evidence of free fluids or colonic contents in the peritoneum. Gentle exploration was done until we identified the abdominal part of the shunt. After following the shunt, we were able to identify the site of perforation in the transverse colon (Figure 2). No active discharge was noticed from the perforation site. We carefully pulled the proximal part of the shunt from the chest. Careful grasping of the transverse colon was done proximally and distal to the perforation. We pulled the shunt from the perforation site, ensuring no stool material leaked into the abdominal cavity. The perforation was around 4x4 mm, which was primarily repaired. Omental fixation of the area with overlying secondary suturing was done. Then we washed the abdominal cavity and applied a drain to the repair site. Post-operatively, the patient was vitally stable, and he was started on gradual oral intake as tolerated.

## Discussion

Shunt migration is defined as the translocation of the whole or a part of the shunt system with and without shunt dysfunction, which occurred in 1 in 1000 patients who underwent a shunt procedure [6].

Headache, gait instability, cognitive impairment, and urine incontinence have been identified as the main symptoms suggesting VP shunt dysfunction. The causes of VP shunt complications are numerous, although they can be broadly classified into three categories: infection, mechanical failure, and functional failure. One of the uncommon effects of mechanical failure is the migration of the VP shunt catheter; this proves to be potentially fatal due to the risk of ascending bacterial meningitis [7]. Various destinations for migration of the distal section of the shunt catheter have been mentioned in the literature, including the umbilicus, colon, stomach, bladder, scrotum, vaginal, anus, thorax, and heart [7].

A rare complication involving bowel perforation may occur in 0.1% to 0.7% of patients following a VP shunt procedure. Most patients with shunt-related bowel perforation typically show symptoms; according to a retrospective review of 139 patients with shunt migration into the bowel, most of them were symptomatic. 74% of cases presented with abdominal symptoms and 21.5% with central nervous system symptoms [8]. Detection of bowel perforation typically occurs within 4-5 months after insertion of the shunt in patients aged 0-1 years old. However, this duration increases with age, reaching a mean duration of 24.8 months across all age groups [9]. Young children who've had a previous infection related to the shunt, restricted abdominal space, or weaker bowel wall are more susceptible to bowel perforation [8].

Children are more likely to have shunt migration mostly due to the shorter travel distance between the caudal and the cranial ends and their growth spurt. A malnourished kid is more vulnerable because there is less subcutaneous fat to hold the tube in place [10]. An increase in the intra-abdominal pressure could also affect the drainage of CSF, which could encourage the extrusion of the shunt through the anal orifice, as seen in this case. However, shunt migration varies among patients depending on the compartment of migration, the direction of migration (cranial or caudal), and the component of the shunt that migrates (proximal catheter, distal catheter, valve, reservoir, or whole shunt system) [10].

In this case, the patient did not present with abdominal symptoms, any signs of peritonitis, localised inflammation, or CNS symptoms other than lethargy and hypoactivity. The VP shunt shows coiling at the site of the transverse colon (Figure 1B), followed by evidence of perforation where it follows the course of the transverse colon and kinking at the splenic flexure (Figure 1A). This might be due to numerous causes, as mentioned earlier, including restricted abdominal space, weaker bowel wall movement, or intestinal peristalsis. Upon extraction of the shunt, the presence of stool was noted within the shunt's tube, which may lead to further complications such as retrograde CNS infections. An infection is considered a fatal consequence of the mechanical displacement of the shunt. If local infection of the shunt tract proceeds, it is best to administer broad-spectrum intravenous antibiotics to prevent the infection from spreading, leading to the CNS [10].

## Conclusions

The ventriculoperitoneal shunt is a surgical procedure commonly used to treat hydrocephalus in patients of all ages. However, this procedure has several complications such as blockage, infection, migration, and detachment from the attached site. It is crucial to be aware of the possibility of VP shunt migration in pediatric patients to identify, manage, intervene, and prevent life-threatening conditions if not treated in time. Therefore, it is recommended to conduct regular radiographic imaging to verify the tube's location and avoid potential complications.
